# CENTERA: A Centralized Trust-Based Efficient Routing Protocol with Authentication for Wireless Sensor Networks ^†^

**DOI:** 10.3390/s150203299

**Published:** 2015-02-02

**Authors:** Ayman Tajeddine, Ayman Kayssi, Ali Chehab, Imad Elhajj, Wassim Itani

**Affiliations:** 1 Department of Electrical and Computer Engineering, American University of Beirut, Beirut 1107 2020, Lebanon; E-Mails: ayman@aub.edu.lb (A.K.); chehab@aub.edu.lb (A.C.); ie05@aub.edu.lb (I.E.); 2 Department of Electrical and Computer Engineering, Beirut Arab University, Beirut 1107 2809, Lebanon; E-Mail: w.itani@bau.edu.lb

**Keywords:** wireless sensor networks, trust, centralized routing protocols, energy efficiency, misbehaving nodes, authentication, identity based encryption

## Abstract

In this paper, we present CENTERA, a CENtralized Trust-based Efficient Routing protocol with an appropriate authentication scheme for wireless sensor networks (WSN). CENTERA utilizes the more powerful base station (BS) to gather minimal neighbor trust information from nodes and calculate the best routes after isolating different types of “bad” nodes. By periodically accumulating these simple local observations and approximating the nodes' battery lives, the BS draws a global view of the network, calculates three quality metrics—maliciousness, cooperation, and compatibility—and evaluates the Data Trust and Forwarding Trust values of each node. Based on these metrics, the BS isolates “bad”, “misbehaving” or malicious nodes for a certain period, and put some nodes on probation. CENTERA increases the node's bad/probation level with repeated “bad” behavior, and decreases it otherwise. Then it uses a very efficient method to distribute the routing information to “good” nodes. Based on its target environment, and if required, CENTERA uses an authentication scheme suitable for severely constrained nodes, ranging from the symmetric RC5 for safe environments under close administration, to pairing-based cryptography (PBC) for hostile environments with a strong attacker model. We simulate CENTERA using TOSSIM and verify its correctness and show some energy calculations.

## 1. Introduction

Wireless Sensor Networks (WSN) are a collection of sensor nodes spatially dispersed to sense and collect data from the environment and collaborate with each other to deliver their readings to a base station (BS) for statistical analysis or merely data collection [1]. Sensor nodes are unattended devices that are severely constrained in terms of processing power, memory size, and energy levels; and thus security and energy consumption are major concerns for any WSN implementation or application. Several security attacks can be launched on a WSN to disrupt its routing scheme, to broadcast false or harmful information, to drain the node battery and thus decrease the network lifetime, among others [2]. There are several methods to detect malicious or misbehaving nodes, and to provide secure routing, which is another critical issue in WSNs, while accounting for energy consumption and hence lengthening the network lifetime. Among these are reputation-based and trust-based methods [3], location isolation [4], and behavior-based techniques [5]. For the proper functioning of these schemes, a secure and efficient authentication scheme is required to validate nodes to each other and to the base station with minimal processing power and data transmission overhead.

Note that the term misbehavior or misbehaving node is used in this paper to reflect a node that willingly or unwillingly, due to a malfunction or defect, interrupts or abuses the functionality of the network in any way possible, except sending malicious packets. Misbehaviors include manipulating protocol-specific parameters, sending through improper neighbors, declaring erroneous data, or even broadcasting or dropping packets.

In this paper, we present a CENtralized Trust-based Efficient Routing protocol with an appropriate Authentication scheme (CENTERA) for WSNs. Utilizing the centralized approach, CENTERA, improving on our previous model CENTER [6], uses the more powerful and more knowledgeable BS to provide a more trusted network environment with more efficient and secure routing paths, while decreasing the load on the severely-constrained sensor nodes.

In CENTERA, the sink BS periodically gathers minimal observations from the individual nodes about the number of packets sent through neighbors and then, it performs several checks and calculations to have a better and more accurate view of the network. The BS approximates the battery life of every node based on its presumed activity and calculates several quality metrics for every node, namely the maliciousness, cooperation, and competence levels. Then, the BS evaluates two trust values for each node—namely Data Trust and Forwarding Trust.

Following the quality metrics calculations, the BS is able to detect several types of bad nodes: a malicious node sending false or illogical information, a non-cooperative node not reliably forwarding the packets of other nodes, or an incompetent node unable to correctly deliver packets to the sink BS, or a malfunctioned/malicious node broadcasting packets. Those “bad” nodes are isolated for a period of time that depends on their history. Thus the sink BS increments the bad or probation level of every node with repeated bad behavior, whereas decrements this level for repeated “good” behavior.

Finally, the BS uses an efficient method to disseminate updated routing information to all the network nodes such that each node knows its uplink nodes to forward their packets to the BS, and its next hop downlink node to forward its own packets through it.

CENTERA provides a trust-based routing protocol while accounting for the severely-constrained sensor nodes batteries and preserving energy in the presence of misbehaving nodes by detecting and isolating them. CENTERA eliminates the power-consuming reputation inquiries and computations required by a distributed approach; nodes are required to send minimal additional information, namely their next hop and a counter (p_counter) for the downlink neighbor (DL) towards the BS and each uplink neighbor, showing the number of packets sent through and forwarded from this neighbor.

For the proper functioning of our routing protocol and the necessary validation of the nodes to each other and to the base station, CENTERA uses a secure and efficient authentication scheme suitable for the extremely limited sensor nodes in WSNs providing acceptable security levels while requiring minimal processing power and data transmission overhead. Based on the target environment, the attacker model, and the sensitivity of the data being collected, CENTERA uses the most appropriate authentication scheme—namely the symmetric key cipher RC5 in case of a safe environment under close administration; or the asymmetric key cipher identity-based encryption—elliptic curve cryptography (IBE_ECC) or PBC in the case of a hostile environment with a strong attacker model. The decision as to which technique to choose and the sizes of the keys and Message Authentication Code (MAC) and their installation in the sensor nodes occurs in the initialization phase prior to launching.

The rest of the paper is organized as follows: Section 2 surveys the previous work in the area of trust-based routing protocols and authentication techniques used in wireless sensor networks. Section 3 presents the system model with the corresponding building blocks, definitions, and parameters. Section 4 explains a list of attacks and misbehaviors and analyzes the methods of detection by CENTERA. TOSSIM simulation verifications and some energy calculations are presented in Section 5. Finally, Section 6 presents some conclusions and future work.

## 2. Previous Work

Wireless sensor networking is a valuable technology to observe and perceive data from the physical world and has an important role in pervasive computing. However, these benefits come with several limitations, vulnerabilities, and risks. Sensor nodes are severely constrained in memory, processing power, and energy resources; and due to their wireless communication and open deployments, they are prone to several security attacks.

### 2.1. Trust and Security in WSNs

In this section, we will discuss the literature related to WSN trust and security. The authors of [1] explain the difference between sensor networks and traditional wireless *ad hoc* networks. For each layer of the protocol stacks, they survey the different issues and technologies for the sensor networks and provide the available solutions for each. They also highlight the open research issues at each layer.

The authors of [7] discuss the challenges in designing a routing protocol for wireless sensor networks and provide a survey of the different available routing techniques. They classify the techniques based on the network structure as flat, hierarchical, or location-based; and based on the protocol operation as query-based, multipath-based, quality-of-service-based, coherent-based, and negotiation-based. For each routing technique, they state its advantages and shortcomings and study the energy and communication overhead tradeoffs.

The authors of [2] consider routing security. They discuss the different attacks and present countermeasures. They analyze the security of the major routing protocols and energy-conserving topology maintenance algorithms for sensor networks. They explain how attacks can be adapted from peer-to-peer and *ad hoc* networks to sensor networks, and present two new attacks: sink holes and HELLO floods.

An energy-efficient routing technique is discussed in [8], where the authors develop and evaluate many techniques to enhance routing based either on energy histograms or solely on localization. They show the network lifetime gains for each technique. Localization is a method that can be beneficial to detect complex colluding nodes attacks.

Several methods are available to detect misbehaving nodes, to lengthen the network lifetime, and to decrease energy consumption while providing secure routing; among these are: reputation- and trust-based methods [3,9], location isolation [4], and behavior-based techniques [5]. Of these, we focus mainly on trust- and reputation-based methods, with some behavior consideration at the BS.

The authors in [9] explain the difference between sensor networks and traditional mobile *ad hoc* networks and survey several trust-based systems in wireless sensor networks. They provide different trust definitions and properties as defined in the literature and state that the definition is application-specific and depends on the methods used to calculate the trust value. They extend the definition of trust to include the sensor data and reliability as a new component and introduce a new trust model that is believed, as per the authors, “to be very robust as it addresses all the drawbacks from the existing approaches.”

The authors in [10] differentiate between the security requirements of WSNs and other networks. They present iTrust, which depends on the presence of monitor nodes to assess the behavior of their neighbors and distribute the trust indices after each session. The authors evaluate their model and show its robustness against different attack scenarios. This method uses the available constrained sensor nodes to assess behavior and distribute trust, instead of fully delegating this burden on the powerful BS.

Trust models are surveyed in [11]. The author explains the difference between security and trust and between reputation and trust. He surveys the methodologies and factors used in the different trust models and states that in wireless sensor networks it is not enough to examine routing messages to infer trust; however, new methods are needed to calculate both communication trust and data trust while keeping in mind that data is sometimes continuous.

In [12] the authors present a security survey on WSNs. They first target the network layer and identify the vulnerabilities and summarize the different defense methods in this protocol layer. Then, they divide the general security issues into seven categories, namely: cryptography, key management, attack detections and preventions, secure routing, secure location security, secure data fusion, and others. In this division, they summarize the different techniques used and point out their pros and cons.

The authors in [13] assure that trust is an important factor for WSNs security schemes and provide a detailed study on trust mechanisms and attacks and countermeasures. They provide a categorization of all trust-related attacks in WSNs. They also analyze the different trust schemes and illustrate the differences and challenges of each. In their work, they provide an extensive literature survey of trust mechanisms used in WSNs and they present open research directions.

In [14] the authors focus on the energy limitations of the sensor nodes and propose a centralized energy-efficient routing protocol for WSNs to reduce energy consumption and thus, increase network lifetime. Their protocol, called Base-Station Controlled Dynamic Clustering Protocol (BCDCP), increases network lifetime and average energy savings by evenly spreading the energy dissipation among all nodes. Although this work does not target trust or security, it is of interest since they highlight the energy saving benefits of a centralized routing scheme, on which we focus our work.

All of the surveyed previous work focuses on distributed methods to calculate trust and reputation and to provide security for the wireless sensor network. However, it is more logical to utilize the centralized approach in a WSN and make use of the more powerful BS to perform these calculations and lessen the burden of the power-consuming reputation inquiries and computations on the sensor nodes.

Several surveys discussed comprehensively the different routing techniques in WSNs [2,7,15–21] presented the most popular research in this field. Table 1 presents a comparison between the most popular routing techniques in WSNs based on common attributes. One thing that can be directly noticed is that out of the whole list, only CENTERA utilizes the centralized approach, which is in harmony with the recommendation of the Open Networking Foundation (ONF), Software Defined Networking (SDN) to use centralization of network intelligence as one of the new norms for networks [22].

To the best of our knowledge and till the time of this writing, CENTERA is the first centralized routing protocol in WSN that incorporates security and trust criteria in the core of the routing decision engine. The different parameters included in Table 1 are explained as follows:

- Classification: Routing protocols are classified based on the network structure into flat-based routing containing nodes with equal roles; hierarchical-based routing containing nodes with different functions; and location-based routing where routes depends on nodes' positions.
- Mobility: Sensor nodes may be considered as stationary or mobile.
- Position Awareness: A sensor node is aware of its spatial position.
- Power Usage: Indicates the power consumption of the whole protocol.
- Negotiation-based: Such protocols initiates negotiation messages prior to data messages in an attempt to eliminate the transmission of duplicate or redundant information.
- Data Aggregation: The option to combine data from different nodes based on some function.
- Localization: The ability to estimate the location of each node.
- Complexity: Given the limited-nature of sensor nodes, applied protocols must be simple.
- Scalability: The protocol must cope with the huge number of typically available sensor nodes.
- Multipath: In an attempt to improve network reliability, some routing protocols utilize multiple paths to route packets at the expense of additional overhead.
- Query-based: In such routing protocols, the destination node queries the network for its required data, and the node with such data replies.
- Centralized/Distributed: In centralized routing protocols, the central node is responsible for the network routing, whereas in distributed protocols, each node has its own routing decision.

### 2.2. Appropriate Authentication Techniques for WSNs

In this subsection, we use some findings from our previous paper [23] specifying the best cipher suitable for the severely constrained sensor nodes in each of the two main categories of the different authentication techniques.

In the first main category, namely the private key cryptography, we deduced that RC5 is the most feasible to be used for WSN scenarios since it strikes the best balance by consuming less energy than most other algorithms while providing better security than algorithms with less energy consumption.

The main drawback of the use of symmetric keys is that both the sender and receiver share the same secret key to perform encryption and decryption. This drawback gets worse in the case where all the nodes in the network share the same key to send their readings periodically to the base station (BS). This approach provides some authentication that the sender is a member of the network, assuming the case of a weak attacker that cannot completely take over a node. Also using this scheme, there cannot be accountability of the exact node that sent false or malicious data into the network. This highly affects trust and reputation schemes and routing protocols, since one malfunctioning node can jeopardize the whole network with no way to point out, punish, or ban such a node.

Another drawback of symmetric key cryptography is that such schemes do not scale well with large numbers of sensor nodes [24]. Thus, in some scenarios, symmetric key cryptography may just not be enough to guarantee the normal and correct functioning of the wireless sensor network; and so the need for other types of encryption is required.

As for the other main category, that is public key cryptography, its use becomes a must in critical networks sensing very sensitive information gathered from hostile environments and it is required to have additional security to be able to point out a malicious or malfunctioning node and isolate it.

From our findings, we deduce that using IBE-ECC or PBC is the most suitable solution for scenarios requiring asymmetric key authentication, which uses the low energy ECC without the challenge of the public key distribution between the nodes, since it simply makes the public key of every entity the same as its name.

In both cases, we noticed two main parameters that affect the limited nature of sensors and thus the lifetime of the WSN: (1) the key size that affects memory and computational overhead requirements; and (2) the MAC size since the MAC will be added to a message, thus increasing the message byte count and as a result, affecting the transmission and reception times and hence the energy requirement.

Accordingly, depending on the sensitivity and nature of the application of the WSN, and the hostility of the environment in which the network is deployed, and prior to launching, the administrator of the network should consider these factors and thus decide upon: (1) the cryptographic technique to be used and (2) the key size and MAC length. By increasing these two parameters, the overhead increases, but the algorithm gets more secure and harder to break. Note that the keys are constructed and installed into the sensor nodes in the Initialization Block prior to network initiation as discussed in Subsection 3.1.

## 3. CENTERA: Our Centralized Trust-Based Efficient Routing Protocol with Authentication

Improving and upgrading our previous model, CENTER, the proposed routing protocol CENTERA implements a centralized trust-based routing protocol with an appropriate authentication scheme for WSNs placing most of the computational load on the more powerful sink BS. Constructing the global view of the network from minimal local information of the authenticated sensor nodes, the BS is responsible for calculating the nodes' trust information and distributing the routing information after isolating the “bad” nodes. CENTERA is divided into eight functional building blocks ensuring the creation of a secure, trusted, and efficient wireless sensor network environment.

### 3.1. Initialization Block

Initially and prior to network launching, the WSN administrator must study the network specifications and needs, including the expected size of the network, the nature of the application, the sensitivity of the data being sensed, and the hostility of the environment where the network will be deployed, among others. Based on the results, the administrator decides on the different parameters for the function of the system.

Among the decisions is the message size that depends on the number of nodes in the network; *i.e.*, any network having less than 256 nodes requires an ID size of 8 bits, whereas the ID size must be greater than that for a network with more than 256 nodes.

Another very important decision is the authentication technique, if any, to be used in the network. So, depending on the hostility of the network and its unattended environment, the administrator chooses to use a strong asymmetric authentication system, like IBE-ECC or PBC, a lighter symmetric system such as the RC5, or not to use any authentication. The administrator decides on the sizes of the used keys and appended MAC, considered x bytes in size. Following this decision, the administrator creates and installs the network master key in all the nodes, in case of the RC5 symmetric cipher choice, or each node's unique private key, in case of the PBC choice.

Other parameters include the hop costs used in Dijkstra's algorithm, the activity period to send an activity/neighbor report, the keep-alive period, the number of allowed probations before a node is considered bad, and the number of periods to decrement the number of probations or level of bad behavior. These parameters will be explained in more details in subsequent subsections. Of course, in this block the administrator installs all the identities, the chosen authentication algorithm, and all the required algorithms and parameters for the proper functioning of CENTERA.

### 3.2. Neighbor Discovery Block

**Figure 1.**
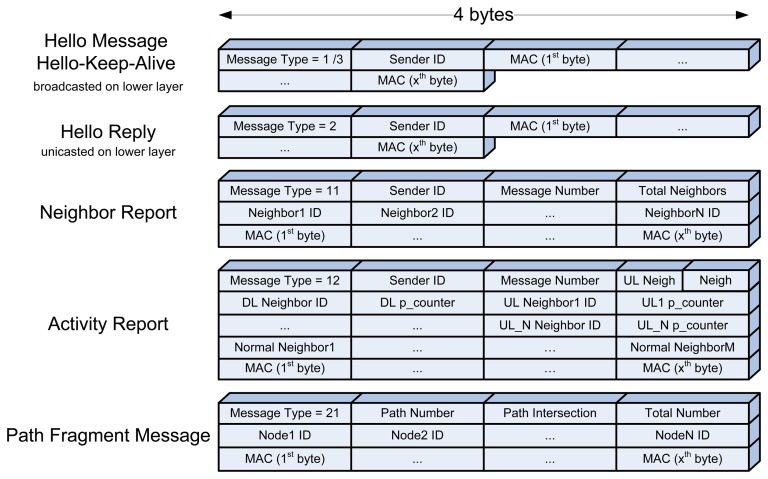
Different Message Formats.

On network bootstrapping or with the introduction of every new node, the Neighbor Discovery Block is activated. In this phase, every node signs and broadcasts a one-hop hello message to introduce itself to its neighboring nodes. The hello message have the following format—{Message Type = 1 [one byte], Sender ID [one byte], MAC [x bytes]} as shown in Figure 1.

Note that x is chosen in the Initialization Block by the administrator based on the network security requirements. Upon receipt of the hello message, each node within radio range, checks the authenticity of the packet, if applicable, by verifying its MAC using the sender's public key (node ID) as the verification key for PBC, or using the symmetric key for RC5, as set by the administrator in the Initialization Block. If the packet is authentic, the receiver node adds the sender in its neighbors list and replies using a signed “unicast” hello-reply packet back to the sender in order to confirm the neighborhood between them. The hello reply message has the same format as the hello message with the Message Type = 2.

Also, every fixed time, set and synchronized by the BS, all nodes broadcasts to its neighbors hello_keep_alive message. The period is set by the administrator in the Initialization Block, and it is chosen to be multiple of times the period of sending the activity reports. This hello-keep-alive message has the exact same format as a hello message with the Message Type = 3, and it is used to make sure that a node still exists in order to keep the BS updated with correct information. Upon receipt of a hello-keep-alive message, the receiving node just refreshes the status of its neighbors, without replying. Note that in case of an authentication scheme set, all types of hello messages must be signed and verified for trusted neighbors' identities.

### 3.3. Node Observation Block

This block is executed when nodes are sending normal messages of sensed data to the BS. As a requirement for this block, each node X keeps a neighbor activity table to store the number of communicated packets to or from each of its neighbors in a certain period, the activity period discussed later. This table contains each neighbor ID, a counter value (p_counter), a flag identifying the uplink (UL) nodes and another flag identifying the downlink (DL) node. In case of an UL neighbor, the p_counter keeps track of the number of packets that node X forwarded from this neighbor through its DL node. In case of the DL neighbor, the p_counter indicates the total number of packets that node X sent and forwarded; in fact the actual number of packets initiated by node X is the difference between the p_counter to the DL neighbor and the sum of the p_counters of the UL neighbors.

Note that the two flags are extracted from the path fragment message sent by the BS (explained shortly). Also note that a node sends its packets to the BS only through its DL and drops any packet received from a node other that its UL nodes.

However, before the Node Observations block can be initiated and every node can start forwarding its data to the BS, the Activity Report Accumulation Block should be initiated for the node to know its downlink neighbor.

Table 2 shows an example of a Node Neighbor Activity Table, where Node X has Node Y as its DL and nodes Z and U as its ULs; whereas node V is just a neighboring node without any interactions with node X. Node X forwarded 4 packets for node Z and six packets for node U. In total node X sent 15 packets through its DL node Y, and thus it initiated five packets.

Note that in case of an authentication scheme set, every normal message communicated in this block must be signed by its initiator to be verified in every hop until the BS. Also, every node forwarding this message encrypts the signature by its own key for its neighbor to verify that it is receiving a packet from an UL neighbor. Thus each node on the path decrypts the signature using the ID of its UL neighbor, and verifies the signature from the source then encrypts the source's signature by its own ID and forwards the packet. Any wrong signature causes the packet to be dropped.

Upon receipt, the BS checks the authenticity of the packet in case authentication is applied, and then increments the number of packets received from the packet source. Then the BS analyzes the packet and if it is found to be malicious, it increments the number of bad packets received from the source.

### 3.4. Report Accumulation Block

The Report Accumulation block is initiated periodically so that every node informs the BS about its neighbors and the packet communication with them, if any. In this block, two types of reports sent by a node to the BS are differentiated; the neighbor report and the activity report.

The neighbor report has a message type of 11 and it is sent by a node every time it has no DL neighbor to send its packets through. This case occurs when the node is first deployed or whenever it is isolated from the network and has no DL in a given period. In the neighbor report, the node sends a list of its neighbors to the BS.

As for the activity report, it has a message type of 12 and is sent periodically by an active node to inform the BS about its neighbor nodes and their corresponding p_counter values, which shows the number of packets sent through or forwarded from this neighbor towards the BS.

Note that the time period of sending a report, the activity period, is a network parameter chosen in the Initialization Block to be of the order of several magnitudes of the period of sending a normal packet containing readings to the BS.

To ensure proper receipt at the BS, each node sending a report in this block, through its next hop neighbor must listen to that next hop for a time t_timeout (a time chosen higher but comparable to the node's time to process and transmit a packet) in order to make sure that the latter has in fact forwarded its packet. If the next hop fails to forward the report during the timeout interval, the node broadcasts its report through all of its neighbors. Every receiving node will send the packet normally through its next hop and performs a similar action.

Note that the overhead incurred is justified in two folds. The first is to assure that such functionally essential reports reach the BS. The second is to help the BS locate and punish the uncooperative nodes. Also note that the broadcasting will not occur frequently in all the nodes, since bad nodes not correctly performing their jobs in sending/forwarding reports will be isolated.

The nodes' neighbor report, shown in Figure 1, has the following format—{Message Type = 11 [one byte], Sender ID [one byte], Message Number [one byte], Total Neighbors [one byte], MESSAGE [(number of neighbors) bytes], MAC [x bytes]}. The message number is needed for nodes to drop multiple copies of the same packet (in case of broadcasting).

As for the nodes' activity report, also shown in Figure 1, has the following format—{Message Type = 12 [one byte], Sender ID [one byte], Message Number [one byte], UL Neighbors [half a byte], Normal Neighbors [half a byte], MESSAGE [(2 + (2 * number of UL neighbors) + (number of normal neighbors)) bytes], MAC [x bytes]}. The message number is needed for nodes to drop multiple copies of the same packet (in case of broadcasting). The message starts with the DL neighbor ID followed by its corresponding p_counter; then each UL neighbor ID is followed by its corresponding p_counter; then a list of the normal [neither UL nor DL] neighbors.

Note that x is chosen in the Initialization Block by the administrator based on the network security requirements. Also note that a node does not send normal reading packets until it receives its UL nodes and its next hop DL from the BS.

In case of an authentication scheme set, similar to the normal messages, the neighbor/activity report communicated in this block must be signed by its initiator to be verified in every hop until it reaches the BS. Also, every node forwarding this report encrypts the signature by its own key for its neighbor to verify that it is receiving a packet from an UL neighbor. Any wrong signature causes the packet to be dropped.

### 3.5. Nodes Analysis and Metrics Calculations Block

After the BS collects and verifies the neighbor/activity reports of all the nodes, the Nodes Attributes and Quality Metrics Calculations Block is initiated. The BS saves the neighbors of each node with the respective counter values as sent by each node and performs a series of checks to detect all discrepancies and misbehaviors in the network. The BS in this block either flags misbehaving nodes as bad or put them on DL probation (indicating a problem with its DL) or UL probation (indicating a problem with an UL node) or neighborhood probation (indicating a problem with a neighbor). A node is put on probation when the BS decides to give the node the benefit of the doubt and give it another chance under different circumstances (different UL or DL). Definitely a node that reaches the maximum allowed number of probations as set by the administrator is flagged as bad; note that the maximum number of probations is a parameter set by the administrator in the Initialization Block based on the network environment and the sensitivity of the exchanged data. Note that if any node is considered bad for any reason, *i.e.*, falsely manipulating counters or sending illogical data or reached limit of probations, the BS neglects its report and included counters in its calculations and checks.

First, the BS verifies neighborhoods of each node. It keeps tracks of nodes removing and then adding their neighbors and flags them as bad after a specific number of unexplained changes. A neighborhood is considered good if it is confirmed by the two neighboring nodes.

After that, the BS validates the reports and counter values. It flags as bad each node declaring forwarding packets through a non-DL neighbor or forwarding packets for a non-UL neighbor. Then the BS calculates the actual packets initiated by each node as the difference between its pcounter to its DL and the rest of the pcounters. If the number of initiated packets is negative, the node is directly flagged as bad.

After those checks, the BS analyzes the nodes and detects potential misbehaving nodes such as packet droppers, lying nodes, colluding nodes, *etc.* It assesses the values of all the counters by comparing them to its received packet numbers and crossing them among all neighboring nodes in the network. So, the BS calculates *Dnb* as the difference between every node's claimed number of forwarded/sent packets and what was actually received by the BS from it. Then the BS calculates the difference *diff* between every node's claimed number of forwarded/sent packets and its DL's claimed number of forwarded packets for this node. The nodes are evaluated as follows:

- if Dnb = 0,
  ○ if diff = 0 the node is good
  ○ if diff ≠ 0 then increment the DL probation of the node and the UL probation of its DL; because of three possible cases, 1- DL maybe lying, 2- the node has a colluding partner down the path dropping its extra undeclared packets, or 3- the node has a clone with dual personality down the path initiating packets in its name
- if Dnb < 0,
  ○ flag node as bad—node is lying since it is declaring less packets than what was actually received by the BS.
- if Dnb > 0,
  ○ if diff ≠ 0 then increment the DL probation of the node and the UL probation of its DL; since node may be manipulating its counters, or its DL is dropping packets, or the link is noisy between the two nodes
  ○ if diff = 0, node is considered as good since its DL has confirmed its declaration at its own responsibility, to be accounted for in later iterations

The BS then approximates the battery life of every node based on its activity estimated by the number of received packets. The BS accounts for the number of transmitted/received packets, signed/verified packets, and encrypted/decrypted signatures. The BS then calculates the different quality metrics for each. Note that all the quality metrics assume values between 0 and 1. The maliciousness is calculated based on the ratio of the bad packets to the total packets received, as follows:

(1) $$\text{maliciousness}(N)=\frac{\sum \text{bad}\;\text{packets}\;\text{received}\;\text{from}\;N}{\sum \text{packets}\;\text{received}\;\text{from}\;N}$$

Using information from all the good packets it received, the sink BS calculates the competence and cooperation of all the WSN nodes. The competence shows the ability of a node to properly deliver a packet to the sink BS and is calculated as the ratio of the packets received by the BS to the packets sent by the node, as follows:

(2) $$\text{competence}(N)=\frac{\sum \;\text{packets}\;\text{received}\;\text{by}\;\text{BS}\;\text{from}\;N}{\sum \text{packets}\;\text{actually}\;\text{sent}\;\text{by}\;N}$$

where the count of packets sent by a node is calculated using its p_counters, which can be checked using the p_counters of the downlink and uplink nodes.

The cooperation shows the willingness of a node N to cooperate and forward packets sent by others towards the sink BS. Cooperation is calculated as the weighted ratio of the number of packets sent by the ULs of N through it over the total number of packets sent by those ULs, as follows:

(3) $$\text{cooperation}(N)=\frac{\sum_{\text{ULs}\;\text{of}\;N}\left(a*\text{pkts}\;\text{rcvd}\;\text{by}\;\text{BS}\;\text{from}\;\text{UL}/\text{packets}\;\text{sent}\;\text{by}\;\text{UL}\;\right)}{\text{count}\;a}$$

Note that (a) is the weight of each uplink node (inversely) related to its maliciousness, as follows:

(4) $$a_{i}=1-\text{maliciousness}(i)$$

The sink BS then calculates two trust values for each node: a Data Trust value and a Forwarding Trust value. The Data Trust of a node n is an indication of the benign nature of the packets of n. It is calculated based on the maliciousness of the node while taking into account the cooperation value (in order to force nodes to cooperate to increase their Data Trust). Data Trust assumes values between 0 and 1 and is calculated as follows:

(5) $$\text{Data}\;\text{Trust}(N)=\frac{\left(1-\text{maliciousness}(N)\right)*\left(\text{cooperation}(N)\right)}{\text{competence}(N)}$$

The Forwarding Trust of a node is an indication of the trust in a node's ability to forward a packet and being confident that the packet will be delivered successfully to the sink BS. The Forwarding Trust is calculated based on the approximated battery level and the competence values of a node, as follows:

(6) ForwardingTrust(N)=(Approximatedbatterylevel(N))*(competence(N))

### 3.6. Bad Nodes Isolation Block

After calculating the quality metrics and trust values for all the nodes, the Bad Nodes Isolation Block is initiated. Any node detected as bad in the previous block will be isolated from the network for a number of activity periods according to its Data Trust (dtrust) level and its banNum value. The banNum is an indicator showing the bad level of the node through time. BanNum is set to one for all nodes and is incremented every time a node is detected as bad.

This block utilizes an effective and efficient method to isolate the detected bad nodes based on its history and current actions according to the following:

- if (dtrust > 0.8) then banRem = 1 * banNum
- else if (dtrust > 0.7) then banRem = 2 * banNum
- else if (dtrust > 0.6) then banRem = 3 * banNum
- else if (dtrust > 0.5) then banRem = 4 * banNum
- else if (dtrust > 0.4) then banRem = 5 * banNum
- else if (dtrust > 0.3) then banRem = 6 * banNum
- else if (dtrust > 0.2) then banRem = 7 * banNum
- else banRem = 8 * banNum

The BS increments the number of successive good periods for every active node not detected as bad nor put on probation in this block. When this number reaches a certain threshold (preset by the administrator in the Initialization Block), the BS rewards the node by decrementing its banNum if it were greater than one, or decrementing its probation number otherwise.

### 3.7. Basic Routing Block

With the bad nodes isolated from the network, the BS starts the Basic Routing Block to find the shortest path for every node towards itself. In this block, the BS first removes the link between neighbors put on probation to check other paths, if any, and really tests the behavior of nodes and pinpoints the bad ones. Then it uses the hop cost set by the administrator in the Initialization Block for all the remaining links and the forwarding trust (*frust*) for each node as the weights for Dijkstra's Algorithm to find the shortest and balanced routing paths of the network. From this block, the BS discovers the UL neighbors of every node and the next hop DL neighbor of every node.

### 3.8. Routing Information Dissemination Block

Finally the Routing Information Dissemination Block is initiated to synchronize the pass number and distribute the routing information to the network sensor nodes. The synchronization is done by sending the number of activity periods (the pass number) as seen by the BS and thus, all nodes will be synchronized.

As for the efficient dissemination of the routing information, the block tries to minimize duplicates information sent over the nodes in order to minimize their communication overhead. This is done by calculating path fragments in the WSN. The path fragment is a path without any bisection. The BS determines the path fragments by first determining all the overlapping paths and then deducing the list of all the path fragments (overlapping by a maximum of one node) and finally uniquely numbering each fragment. This way the overhead of the update messages transmitted through the sensor nodes is decreased to a minimum.

The path fragments, shown in Figure 1, have the following format—{Message Type = 21 [one byte], Path Number [one byte], Path Intersection [one byte of the form path.node], Total Number [one byte], List of N Nodes [N bytes], MAC [x bytes]}.

The path number is the unique number that the BS gives to each path, and the path intersection has the format path.node specifying to which node of which path is the current path connected to. The total number specifies the number of nodes in the current path. When a node receives a path fragment, it may encounter three cases:

- If the node finds itself to be part of the path, it saves the path number together with its location in the current path and the uplink node for that path. In addition, it performs the following:
  - it sets its next hop as the previous location node in the path fragment
  - it adds to its uplink neighbors the next location node in the path fragment
  - it forwards the packet to the next location node in the path fragment
- If the node finds itself to be the intersection byte node (path.node = itself)
  - it adds the new path number to the paths it belongs to, together with the uplink to reach that path (in case there were more paths fragmenting from that path)
  - it adds to its uplink neighbors the next location node in the path fragment
  - it forwards the packet to the next location node in the path fragment
- If the node is not part of the path and it is not itself the intersection node, it checks if it has previously saved the path in the intersection node (path.node). This case occurs when a further-away path fragment is sent through the preceding distributed fragments from the BS; in other words, a closer node to the BS will not appear in the farther path fragment even though it is part of the full path:
  - it adds the new path number to the paths it belongs to, together with the uplink to reach that path (in case there were more paths fragmenting from that path)
  - it forwards the packet to its uplink neighbor to reach the path in the intersection node (path.node)—this uplink neighbor is saved from a previous packet

From this point on and until the next path fragment message, every node sends its periodic reading only through its next hop neighbors and forwards only the packets of its designated uplink neighbors as instructed by the BS. Note that in case of an authentication scheme set, the BS signs each path fragment before forwarding it.

## 4. Attacks and Misbehaviors

This section explains a list of attacks and misbehaviors that can affect the nodes in particular and the network in general, and then analyzes how CENTERA detects them and isolate their effect from the network.

### 4.1. External Attackers

Using any authentication scheme, whether symmetric or asymmetric, the system nodes directly reject any unauthenticated packet coming from an outsider attacker node. Thus, an attacker physically penetrating the system fails to inject any packets into the network. The most harm it can do is some localized noise. Of course this is considered as a simple attacker.

It should be noted here that if the application of the WSN communicates sensitive data, the admin, at the initialization block, may choose to protect the data and force the nodes to encrypt the packet payload, of course at the expense of increases energy overhead. In this subsection, only external attackers are considered. Any external attacker that takes over a node and uses it to launch its attack is considered as an internal attacker discussed in one of the following subsections.

### 4.2. Protocol Specific Attacks

There are several main types of attacks or misbehaviors directly related to CENTERA and its functions. As discussed in Section 3, the BS in CENTERA sets and distributes the routing paths from every node to itself. Thus, the first type that the BS detects is the receipt of a packet on a different path than designated. Knowing that such misbehavior requires the collusion of two nodes, the BS performs the proper checks, and distinguishes a couple of two candidate colluding nodes. The BS puts these nodes on probation and keeps them under surveillance; and isolates whichever set repeating the error.

Another type of protocol specific attacks is a node sending a wrong message format; thus disregarding the rule that in a report the DL should be the first node, followed by the UL (if any) followed by the rest of the neighbors (if any). So any node sending not conforming to this rule is detected as a bad node by the BS. Note that the node may be a malfunctioning node just misplacing its neighbors or a bad node deliberately changing the positions to decrease the trust of its neighbors. It may even be actually trying to send its packets not through its DL and forwarding the packets of a non-UL neighbor. In any case, this node is considered bad and negatively affecting the proper functioning of CENTERA, and thus, it is directly banned by the BS.

A similar kind of misbehavior is the manipulation of counters that results in a negative number of packets initiated by the node. As described in Section 3, the number of packets initiated by a node is calculated as the difference between the pcounter to the DL and the sum of the pcounters to the UL neighbors. If this difference results in a negative value, the node may be malfunctioning or deliberately manipulating its counters and should be banned from the system.

### 4.3. Bad Packet Attacks

This type of attacks is divided into two main parts. The first is when a node is initiating malicious packets intended to harm the nodes or the BS. This type is directly detected by the BS, as it checks all the received packets for any maliciousness. The BS definitely bans this node from the system and isolates its harm.

The second type is a simple attack or misbehavior either by a malfunctioning node or a bad node intending to just flood the network with erroneous packets. Such packets may include an invalid message, an invalid signature, or an unverified signature. This type of attack is directly dropped by the neighboring nodes and thus its effect is localized and minimized.

### 4.4. Packet Number Discrepancies

This type is the most existing attack that is very easy yet very effective. This attack includes packet dropping by uncooperative nodes, nodes lying about their counters, incompetent nodes due to malfunctions or noisy environments.

As discussed in the Section 3, the BS always compares the number of packets received from a node to the number declared by this node; also it compares the number of packets declared to be forwarded by a node to the number declared to be forwarded by its DL. From these comparisons, the BS locates the problem in a link between two nodes; however, it can't specify exactly whether a node is lying or its neighbor is dropping or even there is noise in the link. So, the BS gives these nodes the benefit of the doubt and provides them another chance after removing the link between them. Then, based on a parameter set by the administrator, a node is isolated when the number of maximum allowed probations is reached.

### 4.5. Broadcasting Nodes

Another type of attack is a trial to disrupt the network by always broadcasting packets to all its neighbors. This type is detected and isolated in two stages. First, the effect of the broadcast is locally removed directly since all of its non DL neighbors drop this packet. As for the high transmission rate of the node, this is detected by the BS and decides to put the node on probation or directly isolate it depending on the overhead of the period from what it should be.

### 4.6. Colluding Nodes

This type of attacks is somehow advanced, where two nodes are colluding to disrupt the network or bias it to their advantage. An example of colluding nodes that can really impair the proper operation of the model is the case where an upstream node A is colluding with a downstream node B to drop its extra undeclared packets. This attack aims at decreasing the trust value of benign nodes in the network and banning them. Attacker A sends more packets than it later declares in its activity report, and its benign DL forwards all of its packets; however after some hops down the path, node B drops the extra packets (upon previous agreement) from source A. The goal is to trick the BS into flagging the DL of A as a lying node.

In CENTERA, after the BS detects the difference in the packets sent by A and those forwarded by its DL, and the difference between the packets sent by the UL of B and those forwarded by B, the BS puts both pairs on probation and changes the links between them. In following periods, the colluding nodes persist in the same attempt to disrupt the network, while the other nodes continue operating normally. So, after the number of probations of A and B reaches the maximum allowed, the BS flags them as bad nodes and isolates them from the network. Note that as the number of colluding nodes increases, the BS is faced with more and more misleading reports, until a limit where the logic flips and the BS's decisions start to be inaccurate and erroneous; *i.e.*, the system fails.

### 4.7. Node ID Attacks

This type includes node replication attack and Sybil attacks. In node replication attack, the attacker introduces replicas of one compromised node using its same ID at different locations of the network. Upon the introduction of the replica into a new neighborhood—*i.e.*, connecting to a set of different nodes than the original one, the system may be encountered with two cases. The first is the case where the replica directly starts sending packets without proper introduction with the hello messages, the neighbors will reject its packets as it is assigned as neither their DL nor as their UL by the BS; and thus the attack is directly isolated in this case.

The second case occurs if the added replica kicks off with proper introduction of hello messages, then, the neighboring nodes accept it and send their updated activity report with the replica as a neighbor. Here, there are two cases: 1- if the BS receives two different activity reports containing different neighbors from the same node ID, it directly detects the replication and isolates this node ID, both versions, from the network. 2- If the replicas are more advanced and sending a unified activity report with neighbors from both neighborhoods, the BS detects a neighborhood error (to be discussed in the following subsection) and the node ID will be isolated from the network.

Sybil attacks are where an attacker uses several invented or stolen IDs to sign packets using their IDs and encrypt these signatures by its own ID. This way the attacker injects packets in the name of another node after encrypting its signature by its own ID to appear as a legitimate packet flowing through this path of the network.

Note that, with the incorporation of the Identity based authentication scheme, the attacker can not affect the network without acquiring the master key, which is saved with the admin away from the network, or having access to one or more nodes. Regarding acquiring the master key, it is considered as highly improbable due to the fact that it is saved offline with the administrator. As for the control over nodes, the attacker is considered as a replica with a dual ID. The analysis is similar as before; the BS detects differences in the forwarded and sent nodes, puts nodes on probation, changes paths, and detects and isolates the bad nodes.

### 4.8. False Neighborhood Attacks

This type of attack includes asymmetric neighborhoods in nodes or colluding nodes adding false neighborhood. The first part is when a node A is claiming to be neighbors with node B, and node B is not. This type of misbehavior may be caused by a malfunction or bad nature. In both cases, the BS puts nodes on as much probations as the number of such difference and discrepancies. Thus depending on the majority, the BS is able to detect and isolate such bad nodes.

As for the colluding nodes adding false neighborhood between them, if the nodes are able to forward packets between them in any way, then there is actually a link and neighborhood between them. So, they are evaluated normally in the system depending on their behaviors. On the other hand, if those nodes are unable to forward packets between them, the BS detects the dropped packets between them, when they are associated as UL—DL neighbors. Thus, in any case the BS detects neighborhood attacks without being able to confirm their actual positions. For improved accuracy and localization of such misbehaviors, the administrator may decide to use secure positioning in order to geographically locate nodes and better validate neighborhoods.

## 5. System Simulation

### 5.1. Simulation Setup

In order to evaluate CENTERA and prove its correctness in providing routing information while creating a trusted environment and isolating the bad nodes, we have used the TOSSIM simulator to simulate a grid of Micaz sensors running TinyOS [25]. We used different topologies and different network sizes, a linear network of 30 nodes, a tree of 40 nodes (where each node has three daughters), and a grid network of 5 × 5, 9 × 9, 15 × 15, and 31 × 31 networks.

As for the authentication technique to use, depending on our previous work [23], we choose the asymmetric key cipher technique PBC, which was found out to be the best authentication technique to be incorporated into CENTERA in a hostile environment. We used the TinyPairing library [26,27] and modified the BLS-SS (revised) and the BF-IBE (revised) to have an IBE-SS to sign a message using the private key and an IBE-Encryption to encrypt other node's signature using the private key; where the public key (ID) is used to verify and decrypt respectively.

This way each receiving node can be sure that the source node is indeed the true sender of the packet and the BS can calculate the trust values for the nodes, and properly construct routing paths and detect and isolate malicious/malfunctioning nodes. The attacker model in this case can be assumed to be strong, with the power to take over a node and use it to send packets. With PBC, CENTERA can detect the malicious or compromised node and isolate it completely from the network, thus increasing the network lifetime.

In the network tested, we varied the hop cost between 0, 0.25, 0.5, and 1 and tested the correctness of our protocol and the energy overhead imposed by the additional transmission/receipt and the cryptographic calculations namely signing/verifying and encryption/decryption.

We also assigned several bad nodes to see the effectiveness of our protocol as follows: Node 12 partially non-cooperative dropping one out of every three packets forwarded through it. Node 7 is an outsider node not belonging to the system and node 23 is a partially malicious node sending one bad packet every three packets it sends. Node 17 is declaring sending packets through a non-DL neighbor. Node 3 is incrementing its DL counters and node 19 is incrementing one of its UL counters.

### 5.2. Simulation Results

**Figure 2.**
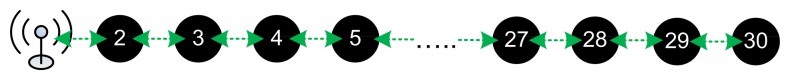
The linear topology.

Initially we tested the correctness of our protocol in the different topologies and sizes. In the linear network, we simulated 30 nodes with the sink BS as the first node. In this type of topologies, shown in Figure 2, each non-border node has only two neighbors and thus the hop cost and weights do not give any difference in the routing path to reach the BS. Also, precautions should be taken in the availability of bad nodes; since any isolation of a non-border node removes a part of the network. In this case specifically and whenever any node has only one DL node to the BS in general, banning a bad node should be based on the type of misbehavior as the requirements and needs of the network (set by the administrator in the Initialization Block). In other words, a tradeoff should be made between misbehavior's effect on the network and the part to be lost in the network. So if the bad node is sending malicious packets for example rendering the whole network useless the node should be banned at the cost of discarding a part of the network; however for simple cooperation errors, the administrator may choose to tolerate this misbehavior at the benefit of keeping the network alive.

**Figure 3.**
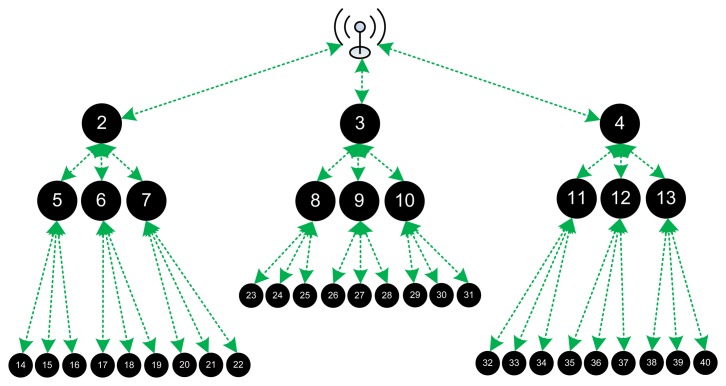
The tree topology.

Another special type of topologies is the tree topology, shown in Figure 3. In the tree topology, each node also has only one path the sink BS, and thus similar reasoning is done as the linear topology case.

**Figure 4.**
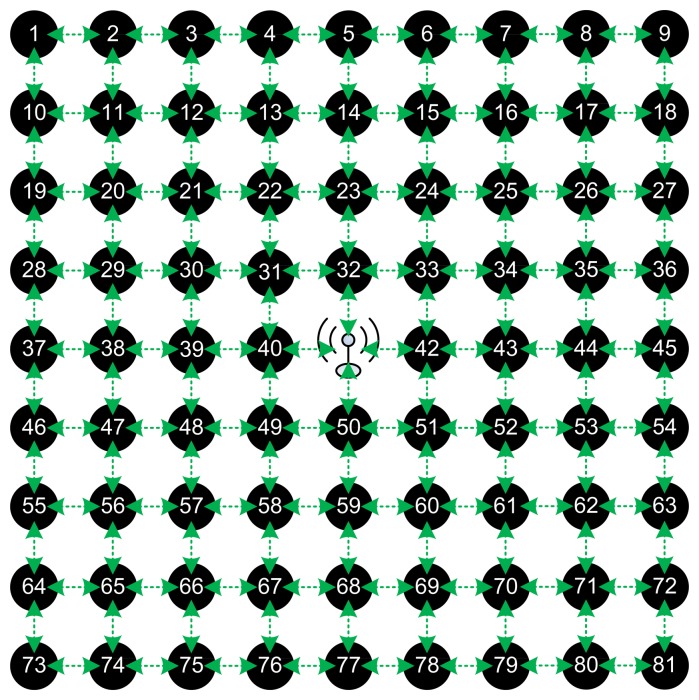
The grid topology (9 × 9).

The hop cost does not change the routing path and a tradeoff should be taken as when to isolate a node and when to bare its misbehavior. We then simulated a general topology, the grid network for different number of nodes ranging from a simple 5 × 5 network to 9 × 9, 15 × 15, and a large 31 × 31 network. In all those topologies, the sink BS is set as the center node and there exists a connection between any two adjacent nodes. We show an example of a 9 × 9 grid topology in Figure 4, where the green arrows show radio range between the nodes. Initially, the different blocks of CENETRA execute correctly and the BS is able to create shortest paths routing information and distribute such information to the different nodes.

**Figure 5.**
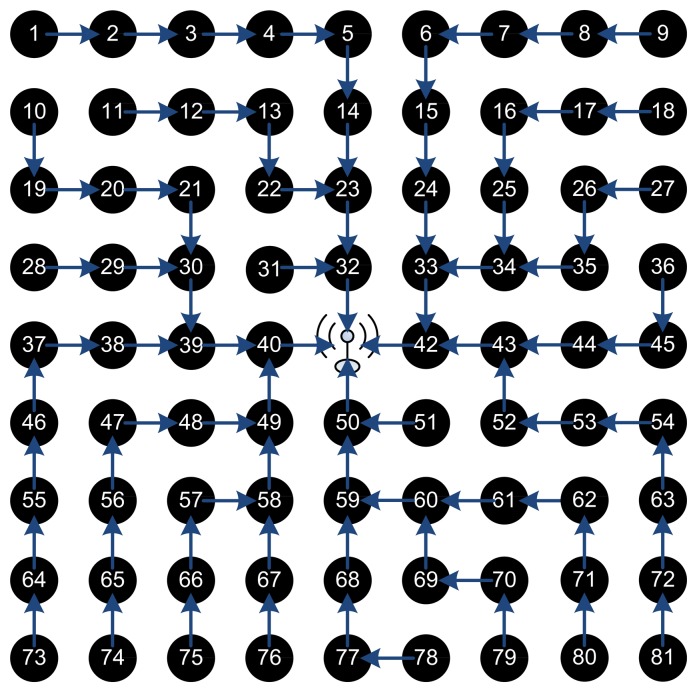
Initial routing paths in the 9 × 9 grid topology (hop cost = 1).

Figure 5 shows the network initial routing paths of the different network nodes with hop cost equal to 1. Note that as the time passes and the battery lives of nodes depreciate the paths change to distribute the load in a balanced way and depreciate all nodes equally.

Changing the hop cost in Dijkstra's algorithm from one to zero gives the possibility to add longer paths from nodes. For hop cost equal to one the BS chooses the most efficient path from the set of shortest paths for each node, while keeping the network relatively balanced. For hop cost equal to zero, the choice depends solely on the ftrust value (incorporating the battery life and competence) totally neglecting the hop count in the decision. Thus, it chooses the path with the least ftrust at the expense of increasing the overall communication energy overhead in the network as a whole. Also, with hop cost equal to zero, the routing paths are very dynamic and highly changing every period.

**Figure 6.**
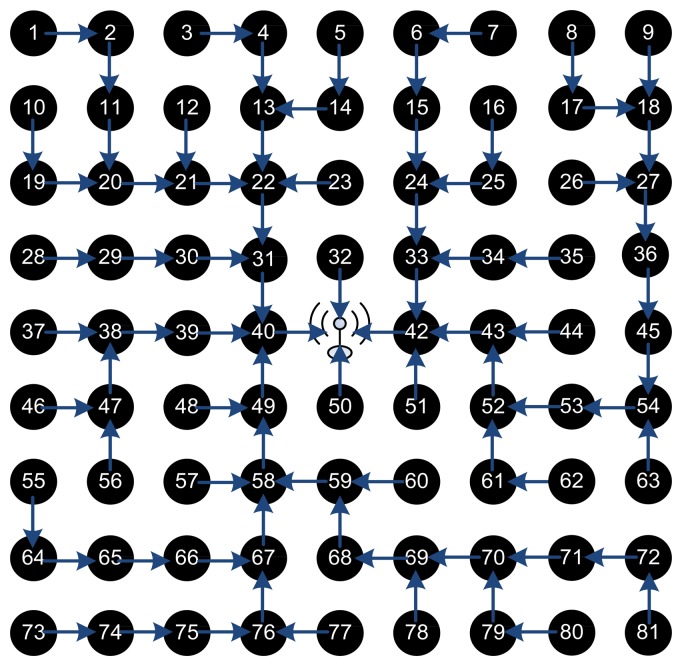
Routing paths with hop cost equals zero.

Figure 6 shows the routing paths of the 9 × 9 network with hop cost equal to zero. In this pass, the BS approximated battery levels of nodes 32 and 50 are very low, of node 42 is 60%, and of node 40 is 70%. It is obvious how the protocol almost fully depleted two nodes while the other two nodes are still good on power. Also the paths are very long and most nodes depend on one BS neighbor (50 nodes are forwarding their packets through node 40!) causing a bottle neck, fast depletion and a higher probability of dropped packets. Consequently, in our tested grid topology, removing the hop cost and depending solely on the ftrust is not a good option to consider.

**Figure 7.**
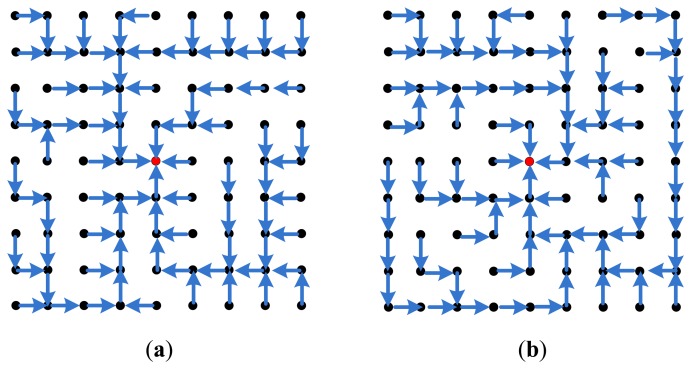
(**a**) Routing paths with hop cost equals 0.5; (**b**) routing paths with hop cost equals 0.25.

Figure 7 shows the routing paths with hop cost equal 0.25 and 0.5. Similar results and analysis are seen that as the hop cost decreases, the routing paths are longer and more dynamic. It is clear that as the hop cost increases the paths are more symmetrical and balanced.

Table 3 shows the load on the BS neighbors in this specific period and assures our conclusion that as the cost of the hop increases the routing paths are more balanced and less dynamic every period. It should be noted that longer and unbalanced paths have the advantage of depleting the whole network together, which could be useful in some types of irregular networks. This comes at the expense of increasing the overall network communication energy consumption and longer paths, which by itself could pose the risk of higher packet dropping rate. Similar results are found in smaller topologies such as 5 × 5 and larger topologies such as the 15 × 15 grid.

As for the 31 × 31 topology, it contains 961 nodes and thus requires more than one byte to account for the node IDs. So, we doubled the message size and simulated the network again. The protocol proved to be correct even for such large networks, giving a balanced and shortest path routing for all nodes in the network.

Following we discuss the detection and isolation of bad misbehaving nodes for which we assigned several bad nodes as discussed in the simulation setup. First, node 12 was set to be a partially non-cooperative node dropping one out of every three packets forwarded through it. In the sixth pass, that is the first activity period after the BS distributed the DL and UL information to all the nodes, node 12 is put on UL probation and its UL node 11 is put on DL probation. Then the BS changes the link between the two nodes to detect which node is misbehaving and set node 3 as an UL of node 12. In the following activity report period, pass 12, the BS found a difference in the counters of node 3 and node 12. Thus in giving node 12 another UL probation, with the maximum allowed probation set to 1, node 12 is set as a bad node, and thus isolated from the network for the next three periods, since its dtrust value is 0.67 and its banNum is still 1, so as per the banning system, the banRem is set to three activity periods and the banNum is incremented to 2.

Thus, as described earlier, when the BS detects a difference in what nodes are claiming to have sent/forwarded through/for each other, it gives them another chance and changes the link between them; since this may be due to a noisy link, lying node, or uncooperative node. As seen in this example, even a node 12 partially dropping packets can be detected by the BS, if it persists on its bad actions. Note that the decreasing the probation limit increases the decision to ban a node at the expense of false positives. On the other way, increasing the probation limit gives the misbehaving node more time to exploit the network and drop its neighbors' packet.

Then node 7 is an outsider attacker node not belonging to the system and node 23 is a partially malicious node sending one bad packet every three packets. The results showed that node 7 is isolated from the system as it does not have the required key to sign its packets, and thus all of its packets are dropped by its neighbors 6, 8, and 16. As for node 23, the BS detects each malicious packet and set it as a bad node and then isolated for 2 periods, since its dtrust value is 0.75 (sending only one bad packet out of four in the first period). Definitely, in subsequent periods, node 23 is isolated more and more for every additional malicious packet sent.

Table 4 details the different values and describes the banning process. In the first activity period, the BS has just received the neighbor report from the nodes, and thus there is still no data to assess the nodes. In the second activity period, the BS has received five nodes from the source node 23 out of which one is detected as a malicious packet with harmful content; so directly the BS flags node 23 as bad without any probation. Then the BS calculates the rest of its traits and values, resulting in banning node 23 for two periods and increasing its banNum to 2; which is clear in the table. Note that banNum acts as a history for bad activity. In the end of the fourth period, the isolation time has ended and the BS includes the node into the network again. However, in the fifth activity period, the BS detects a malicious packet again, and thus the BS decides now to isolate the node for three periods. Note that, since node 23 is a partially malicious node and it is sending a small percentage of bad packets, its dtrust is not very low, and thus its banning period is increasing slowly one time after another. This would have been much more aggressive had the node been totally malicious.

This example shows two main features of our model, 1- the network inherently isolates outsider nodes using the strong yet efficient authentication scheme, and 2- the BS directly recognizes the bad, misbehaving node by detecting its sent malicious content even if at a low rate. In this example, the BS isolates node 23 for two periods only as an initial countermeasure since node 23 has not previous bad actions. After the banning period ends, BS tests node 23 again, however this time node 23 has a history and thus when node 23 repeats its malicious activity, it is banned for five periods. This continues by increasing the banning periods before rechecking the node by giving it an additional chance; and anytime the node stops its bad deeds, its banNum starts decreasing until it is considered as a good node again.

In the following, we show some misbehavior constituting counter manipulations. Node 17 is claiming to send packets through a non-DL neighbor and thus, the BS directly detects it as a bad node for that. As the dtrust of node 17 is still equal to 1 and it has no history of bad actions, it is isolated for one period initially, the number which increases as the misbehavior persists. Note that the reason behind this misbehavior may be a malfunctioning node or a bad node trying to delude the BS into considering some good node as bad; however, in any case, this type of misbehavior should be directly stopped as it affects the correct operation of the system.

Then, we added two misbehaving nodes, node 3 incrementing its DL counters and node 19 incrementing one of its UL counters. At pass 6, the BS detects the discrepancies and put node 3 on DL-probation and its DL, node 4, on UL-probation; it sets the DL of node 3 as node 12. As for node 19 it has no ULs for this period and it did not do anything wrong so far. In pass 12, node 3 is detected as bad since it took a second DL probation. In this pass, node 19 manipulated its counters for its UL (node 10), and thus node 19 is put on UL probation and node 10 DL probation. In pass 18, node 19 is isolated. We also got similar results when repeating the simulation with node 3 decrementing its DL counters and node 19 decrementing one of its UL counters.

Thus, CENTERA can detect any node trying to manipulate its counters due to malfunctioning or due to the intention to hurt other nodes and cause them to be banned. In either case, the BS can after some checks and analysis isolate the exact misbehaving node.

To further analyze the benefits of authentication in CENTERA, we take a look at two attackers on the network, nodes A and 4. It is directly noticed that the outside attackers are isolated completely from the network in both cases. The strong attacker took over node 4. It tried to impersonate other nodes, but this is impossible without the private key that is the identity of the node. So, the attacker started using node 4 to send bad packets into the network. The BS updated the routing paths of the nodes such that node 4 is totally isolated from the system. As for the attacker node A, without proper authentication, it is directly neglected by the all the nodes in the system, and if it used the same authentication technique, the BS directly updates the routing paths to neglect this outsider.

**Figure 8.**
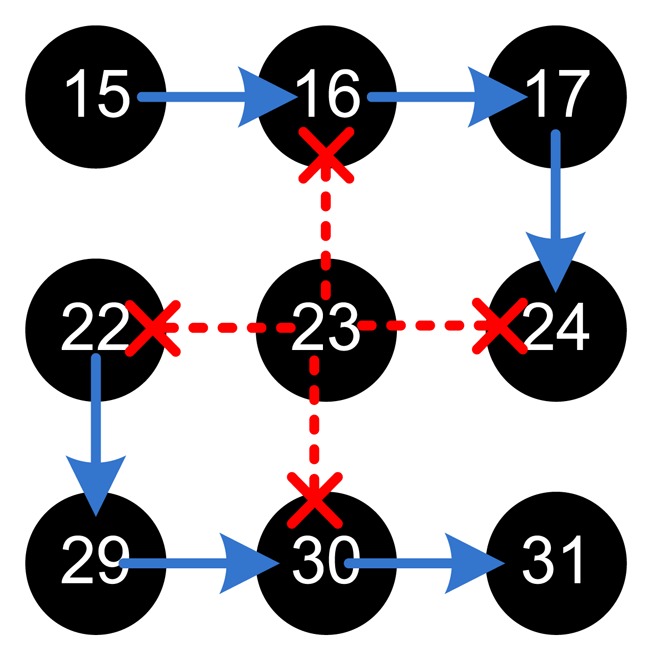
The isolation of the broadcasting node 23.

We finally tried a broadcasting node, node 23 that is trying to broadcast packets through nodes 14, 22, 24, and 32, either due to a malfunction or in order to disrupt the whole network. However, it is clear, from the snippet of the topology shown in Figure 8, how CENTERA forced nodes 14, 22, and 24 to drop the packets from 23 because they are not the DL of node 23 as indicated by the BS. Only node 32 is forwarding the packets of node 23. Thus here we can directly see the first benefit of CENTERA in preventing broadcast storms that can increase the noise levels in the network and affect the network functionality and lifetime. Also for any discrepancies declared in its pcounters, node 23 is punished and isolated as the cases stated above.

Thus, as deduced from the simulations, after the first time period, each node starts acting per its nature. This directly gets reflected in the routing paths that now avoid the bad nodes. The routing paths are updated to avoid the “bad” nodes and pass only through “good” nodes. So, all of the bad nodes are isolated and no other node is forwarding their packets.

### 5.3. Energy Calculations

In order to evaluate the energy consumption of CENTERA, we simulated an average sized grid topology of 9 × 9 nodes and extracted the bytes transmitted/received and those used under cryptographic calculations. In both cases we differentiate the initial activity period—where the nodes are not yet informed about their DL neighbors, and the remaining subsequent periods. We show the total bytes in the whole network, the average bytes per node, and the worst case in each activity period (the node that endured the maximum energy consumption).

Note that in our simulation, we choose the activity period to be 6—that is the nodes sent their activity reports and the BS update the routing paths every five periods of sending a normal message.

Table 5 shows the number of bytes transmitted and received by each block of our model running without any authentication technique. We show the numbers in four passes where activity/neighbor reports are sent, representing the full network, the average bytes per node, and the worst case.

From Table 5, it can be seen that the hello messages occurred in passes 2 and 20, which is normal since pass 2 is the initial period where nodes send hello and help reply messages to get acquainted, whereas pass 20 is the third activity period and in our simulations this is the time to send hello keep-alive messages. The network as a whole has communicated 1328 bytes of those messages, averaging to 16.6 bytes per node; and the worst case was node 42, which actually received all of the hello replies from all of its neighbors. Note that, some nodes may not receive the total number of hello replies due to noise or collisions, but CENTERA's hello scheme is resilient to such effect. As for pass 20, the network communicated a less number of 728 bytes due to the fact that hello keep-alive messages don't require a reply. The average was 10 bytes per node, which is logical since every node has to broadcast one keep-alive message and receive as much keep alive messages as the number of its neighbors; and each message contained just two bytes.

As for the reports, in the initial phase the reports consist of neighbor reports only. The network communicated 130,514 bytes averaging around 1631 bytes per node; with the worst case being 1823 bytes. This number of bytes may seem a bit high, however this occur only in the initial phase where the nodes still do not have downlinks and, thus, they broadcast their neighbor reports to reach the BS. So, it is considered as a startup overhead that is insignificant in the life time of the network.

In subsequent phases, the reports consists of the activity report, larger in size, but smaller communication overhead, since nodes forward them through their DLs to reach the BS. In these phases the average node communicated as low as 85 bytes of activity reports, with the worst case being 564 bytes for node 42; normal for a direct neighbor of the BS.

The subpaths sent by the BS to disseminate the routing paths and teach every node its DL and ULs, show close number of communicated bytes in both phases, initial and subsequent. Averaging around 45 bytes per node, the overhead is very minimal for one of the main steps in the model. Therefore the total communication OH per node from CENTERA's blocks, when authentication is not added, reached 1691.9 communicated bytes in the initial phase, and settled at around 130–140 communicated bytes per node in subsequent phases.

**Figure 9.**
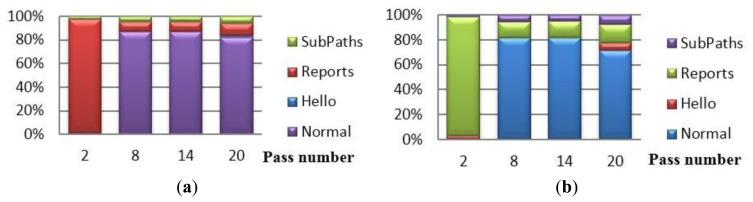
(**a**) Communication overhead without authentication; (**b**) communication overhead with authentication.

Considering the normal date packet containing sensor readings constitute of 28 bytes (as set by TinyOS), the communication overhead of CENTERA range from 12% in normal periods to 17% in periods where the keep-alive message is sent. Note that these percentages are calculated in our simulations where the activity period is take to be as low as 6. For relatively stable networks the activity period may be chosen by the administrator to be 50, decreasing the communication overhead to less than 2%. Figure 9a, shows the different percentages of the communication energy dissipated by the blocks of CENTERA and the normal data packets exchanged. It is obvious that, with the exception of the first activity period, CENTERA is adding little overhead (12% to 17%) to the normal function of the WSN.

**Figure 10.**
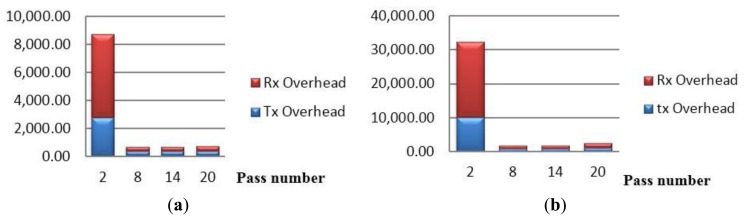
(**a**) Transmission energy without authentication in μJ; (**b**) transmission energy with PBC authentication in μJ.

Using the energy estimations found in [28], where the energy consumed in MICAz for transmission is 0.6 μJ/bit and for receipt is 0.67 μJ/bit, Table 6 contains the exact energy consumed per block per period. Figure 10a shows the proportion of the overhead incurred by CENTERA between the transmission energy to that of the reception energy. Two things can be noticed, (1) the reception energy is slightly higher than transmission energy and (2) the energy consumption spikes at the first period to around 8 mJ and then averages to around 650 μJ/period for the remaining network lifetime. Table 6 shows that the energy dissipated to transmit and receive normal sensor packets is around 4000 μJ to 4500 μJ/period. Thus, the communication energy overhead is minor when compared to the normal functioning of the sensor network.

Note that, increasing the activity period from 6 to 50, for instance, decreases the overhead even further, as the number of normal packets sent per period is multiplied by 8 and thus the overhead ratio sinks from 650/4500 ≈ 14% to around 1.7% overhead.

As for the system with a proper authentication scheme incorporated, the overhead imposed by CENTERA will be higher due to the extended messages communicated and the cryptographic technique used. We include the Identity Based—PBC, due to its lightweight processing, short signature (160 bits) and most importantly zero energy and storage to communicate and store keys of every node. This is a direct advantage gained from using Identity based encryption.

Tables 7 and 8 repeat the analysis of Tables 5 and 6 in showing the overhead of CENTERA when PBC is incorporated. Similar to the previous results, the overhead is still low as compared to the normal packets communicated by the sensors, as shown in Figure 9b. The overhead now remains at around 18% and rises to 28% in periods where keep alive messages are communicated. This raise is partly because of the additional transmissions and receipts incurred on the network, but majorly due to the fact that in such activity periods, there will be one less period of sending normal packets. Note that, similar to previous analysis, the overhead decreases drastically by increasing the activity period from 6 to 50 from 18% to around 2% and from 28% to around 2.8%.

This stresses the importance of correctly setting the different parameters, and specifically the activity period, which specifies the speed of updating the network and detecting errors at the expense of spending more energy.

Also Figure 10b shows similar results to that of Figure 10a, with the difference that here the energy consumption spikes at the first period to around 30 mJ and then averages to around 1.7 to 2 mJ/period for the remaining network lifetime. This increase is due to the added authentication bytes communicated by the nodes. Table 8 shows that the energy dissipated to transmit and receive normal sensor packets is around 6 mJ to 8 mJ/period. Thus, the communication energy overhead is still minor when compared to the normal functioning of the sensor network.

In Table 9, we show the effect of the cryptographic techniques to perform the required authentication to secure the WSN in the most hostile environments. We divided the study as per the number of bytes signed and verified by each node to determine the initial sender of the packet. Also there is the number of bytes where the subsequent nodes have to encrypt and decrypt the source's signature in order to verify the direct hop-by-hop forwarder of the packet.

Note that in the initial phase the major overhead is due to the verification of the broadcasted neighbor reports, which loaded the network by 132,288 bytes to verify, averaging 1653.5 verified bytes per node. The total number of bytes processed by cryptographic functions reached 135,660 bytes in the network averaging 1695.75 bytes per node in the initial phase. In subsequent phases, this number dropped to around 88,000 bytes in the whole network, averaging to around 1100 bytes per node. Note that the last period, where there is one less period to send normal packets, the total is just 70,664 bytes to authenticate, the thing that clearly shows that the majority of the processing overhead is spent authenticating the normal sensor messages.

One thing that can be noticed is that the worst case in the normal phases is much higher than that of the initial phase. This is clearly described by the absence of normal packets in the initial phase. This gives an indication of the huge load that the closer nodes to the BS have to endure due to the nature of such networks.

The majority of the cryptographic overhead is due to the normal packets communicated and not due to the blocks of CENTERA. This conforms to the previously gathered results where we noticed that the additional number of packets sent by CENTERA is in the range of 12% to 17% for the chosen activity period.

To further show the advantage of authentication in a trust based system for energy efficiency, we introduced into our system a broadcasting attacker node while changing its identity and calculated the cumulative number of bytes exchanged until each pass.

In this scenario, node 11 broadcasts normal packets to its neighbors at a high rate while changing its identity, in order that its packets are forwarded by its neighbors into the system. As explained previously, CENTERA forces nodes to only forward the packets of their designated UL neighbors.

**Figure 11.**
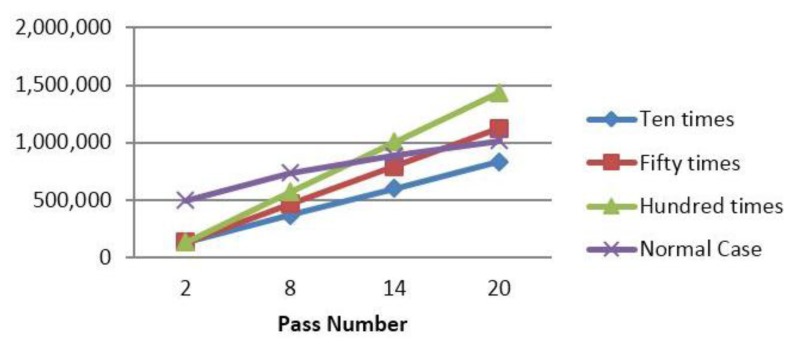
Cumulative number of exchange bytes in the network up to each pass.

We repeated this scenario while changing the broadcasting rate of node 11, from ten times the normal rate of normal packets, to 50 and 100 times. Table 10 shows the cumulative number of bytes exchanged for the whole network of 81 nodes, the average number of bytes per node, and the worst case, in each broadcasting rate and compares them to the normal case of CENTERA with authentication. It is clear from the table how the overhead of the authentication is offset by the broadcasting attacker in as low as three and four passes of the system for the 100 and 50 times broadcasting rate cases. Also in the low rate of ten times broadcasting rate, the overhead is closing up in four passes in the table. Figure 11 displays the network exchanged bytes and it is clear how the authentication overhead is overcome very quickly by one attacker.

Note that in CENTERA this attack will be limited in time and space. In CENTERA and as a direct benefit of authentication, node 11 is unable to deceive its neighbors by using their UL node ID and thus those neighbors drop the packets of the attacker; the attack is limited in space. In the next pass, as the packets are authenticated, the BS detects the high sending rate of node 11, and isolates it to end its negative effect from the system; the attack is limited in time.

Finally, we calculate the network life-time in terms of the first node dead (FND) and the residual energy after multiple rounds of data exchanged. As we couldn't find exact energy calculations of compute one byte of PBC authentication, we consider it to be x% of the energy to receive one byte (as it is higher). We vary x between 10%, 50%, and 100%. Note that it is well known that the energy to compute is much lower than the energy to transmit/receive a byte, thus our chosen values of x are all high assumptions to check the worst case of the energy life-time.

We consider that each sensor node has a small battery of 15,000 J and perform the life analysis as follows. Table 11 shows the network life-time calculations while varying the value of x between 10%, 50% and 100%. The results are the average energy consumption in the first pass and the average in the following passes in mJ. Also it shows that the first node dies after over 203,073 passes when x = 10% and over 83,482 passes even when considering the energy to compute one byte equal that to receive one byte.

**Figure 12.**
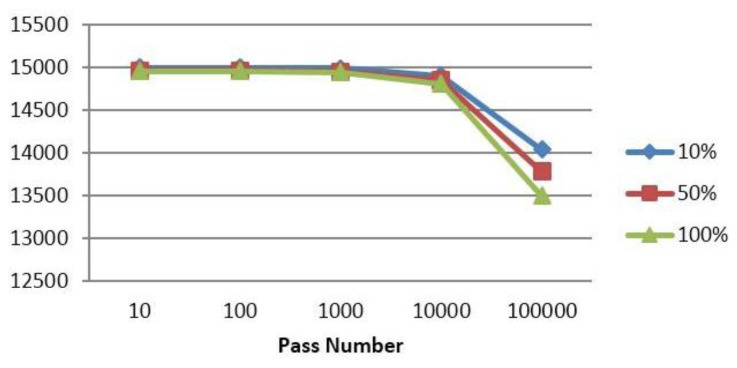
Average node residual energy in J.

Figure 12 shows the average residual energy in every node starting with a full 15,000 J battery at multiple of ten passes.

It shows the low energy consumption of CENTERA, since even when considering the energy to compute one byte equals that to receive a byte, the average residual energy after 1,000,000 passes is still 13,497 J which is around 90% of the battery charge; whereas the battery is still over 93.5% assuming x = 10%.

**Figure 13.**
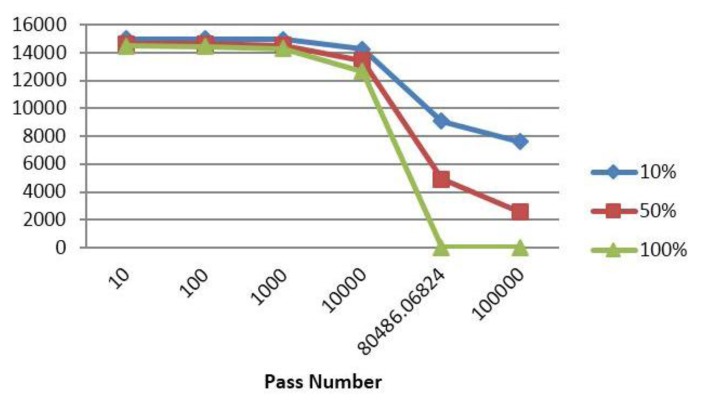
Worst case residual energy in J.

Figure 13, shows the residual energy for the most active node, or closest to the BS. It can be seen that even in the most unfair assumption of x = 100%, the first dead node is seen at over 80,000 passes. Also, the figure shows that assuming x = 10%, the residual energy in the worst case is a bit over 50%.

## 6. Conclusions and Future Work

In this paper, we presented CENTERA, a CENtralized Trust-based Efficient Routing protocol with an appropriate Authentication scheme for wireless sensor networks (WSN), which periodically sends readings and sensed data to a powerful sink BS. CENTERA provides secure routing and a trusted network where the bad nodes are isolated and their attacks eliminated. We classified different types of bad nodes, some of which are malicious, incompetent, non-cooperative, broadcasting, outsider, and impersonating nodes that affect the routing functionality of the network.

CENTERA utilizes the centralized approach, where the more powerful BS periodically accumulates simple counter observations from the sensor nodes and decides on the network topology and routes, and isolates the bad nodes. The BS calculates several quality metrics and two trust levels of each node and uses an effective banning system to isolate the different bad nodes from the network. Also, CENTERA uses a very efficient method to distribute the routing information to every node. The nodes forwards only through their next hop DL neighbor and forward the packets of their UL neighbors, only as indicated by the BS and drops any other packet.

Based on its target environment, CENTERA uses a secure and efficient authentication scheme suitable for the severely constrained nodes; the authentication technique may be the symmetric key cipher RC5 in case of a safe environment under close administration, or the asymmetric key cipher PBC or IBE-ECC in case of a hostile environment with a strong attacker model.

We simulated CENTERA using TinyOS and proved its correctness in providing secure routing information through trusted paths. Also, in CENTERA, some nodes were put on probation to observe closely while bad nodes were isolated for a specific time depending on their history, and then given another chance to try to improve.

Our future work will focus majorly on further detailing and enhancing the BS analysis algorithm to more efficiently perform all the complex tasks it is entitled to do. We will also perform additional simulations on irregular network topologies to further analyze the effect of hop cost. In addition, we will check the inclusion of secure positioning or other positioning system to better validate nodes' neighborhood and detect colluding nodes.

## Figures and Tables

**Table 1.** Comparison among Most Popular WSN Routing Techniques.

	**Classification**	**Mobility**	**Position Awareness**	**Power Usage**	**Negotiation-Based**	**Data Aggregation**	**Localization**	**Complexity**	**Scalability**	**Multipath**	**Query-based**	**Centralized/Distributed**
**CENTERA**	Flat	No	No	Very Limited	No	No	No	Low	Good	Possible	No	C
**SPIN**	Flat	Possible	No	Limited	Yes	Yes	No	Low	Limited	Yes	Yes	D
**Directed Diffusion**	Flat	Limited	No	Limited	Yes	Yes	Yes	Low	Limited	Yes	Yes	D
**Rumor Routing**	Flat	Very Limited	No	N/A	No	Yes	No	Low	Good	No	Yes	D
**GBR**	Flat	Limited	No	N/A	No	Yes	No	Low	Limited	No	Yes	D
**MCFA**	Flat	No	No	N/A	No	No	No	Low	Good	No	No	D
**CADR**	Flat	No	No	Limited	No	Yes	No	Low	Limited	No	No	D
**COUGAR**	Flat	No	No	Limited	No	Yes	No	Low	Limited	No	Yes	D
**ACQUIRE**	Flat	Limited	No	N/A	No	Yes	No	Low	Limited	No	Yes	D
**EAR**	Flat	Limited	No	N/A	No	No	No	Low	Limited	No	Yes	D
**LEACH**	Hierarchical	Fixed BS	No	Maximum	No	Yes	Yes	High	Good	No	No	D
**TEEN & APTEEN**	Hierarchical	Fixed BS	No	Maximum	No	Yes	Yes	High	Good	No	No	D
**PEGASIS**	Hierarchical	Fixed BS	No	Maximum	No	No	Yes	Low	Good	No	No	D
**MECN & SMECN**	Hierarchical	No	No	Maximum	No	No	No	Low	Low	No	No	D
**SOP**	Hierarchical	No	No	N/A	No	No	No	Low	Low	No	No	D
**HPAR**	Hierarchical	No	No	N/A	No	No	No	Low	Good	No	No	D
**VGA**	Hierarchical	No	No	N/A	Yes	Yes	Yes	High	Good	Yes	No	D
**Sensor aggregate**	Hierarchical	Limited	No	N/A	No	Yes	No	Low	Good	No	Possible	D
**TTDD**	Hierarchical	Yes	Yes	Limited	No	No	No	Moderate	Low	Possible	Possible	D
**GAF**	Location	Limited	No	Limited	No	No	No	Low	Good	No	No	D
**GEAR**	Location	Limited	No	Limited	No	No	No	Low	Limited	No	No	D
**SPAN**	Location	Limited	No	N/A	Yes	No	No	Low	Limited	No	No	D
**MFR**	Location	No	No	N/A	No	No	No	Low	Limited	No	No	D

**Table 2.** Node Neighbor Activity Table at Node X.

**Node**	**P_Counter**	**UL**	**DL**
Node Y	15	No	Yes
Node Z	4	Yes	No
Node U	6	Yes	No
Node V	0	No	No

**Table 3.** Load on the BS neighbors with respect to hop cost.

**Hop Cost**	**Node 32**	**Node 40**	**Node 42**	**Node 50**
1	12	26	25	13
0.5	6	29	0	41
0.25	1	0	30	45
0	0	50	26	0

**Table 4.** Different Values of node 23 at BS every period.

**Activity Period**	**1st**	**2nd**	**3rd**	**4th**	**5th**
DL	-	32	-	-	32
Received at sink	-	5	-	-	4
Node received	-	45	-	-	92
Node forwarded	-	50	-	-	96
Bad Received	-	1	-	-	
Maliciousness	-	0.2	-	-	0.2
Competence	-	1	-	-	1
Cooperation	-	1	-	-	1
Ftrust	-	0.3	-	-	0.3
Dtrust	-	0.8	-	-	0.8
BanRem	0	2	1	0	3
BanNum	1	2	2	2	3
Probation	0	0	0	0	0
Bad	0	1	0	0	1

**Table 5.** Bytes Transmitted and Received without Authentication.

**Pass**		**Hello**	**Reports**	**SUB**	**Total OH**	**Normal**	**TOTAL**
2	Network	1328.00	130,514.00	3510.00	135,352.00	0.00	135,352.00
	Av. Per Node	16.60	1631.43	43.88	1691.90	0.00	1691.90
	Worst Case (42)	20.00	1823.00	186.00	2029.00	0.00	2029.00

8	Network	0.00	6856.00	3574.00	10,430.00	71,680.00	82,110.00
	Av. Per Node	0.00	85.70	44.68	130.38	896.00	1026.38
	Worst Case (32)	0.00	516.00	209.00	725.00	5264.00	5989.00

14	Network	0.00	6856.00	3479.00	10,335.00	71,680.00	82,015.00
	Av. Per Node	0.00	85.70	43.49	129.19	896.00	1025.19
	Worst Case (50)	0.00	784.00	113.00	897.00	7952.00	8849.00

20	Network	728.00	6856.00	3916.00	11,500.00	53,760.00	65,260.00
	Av. Per Node	9.10	85.70	48.95	143.75	672.00	815.75
	Worst Case (32)	10.00	564.00	240.00	814.00	4284.00	5098.00

**Table 6.** Transmission Energy in (μJ) without Authentication.

**Pass**		**Hello**		**Reports**		**SUB**		**OH**		**Normal**	

		**Tx**	**rx**	**tx**	**rx**	**tx**	**rx**	**tx**	**rx**	**tx**	**rx**
2	Network	3494.40	3216.00	207,782.40	467,531.36	9772.80	7900.64	221,049.60	478,648.00	0.00	0.00
	Av. Per Node	43.68	40.20	2597.28	5844.14	122.16	98.76	2763.12	5983.10	0.00	0.00
	Worst Case (42)	48.00	53.60	2822.40	6619.60	556.80	375.20	3427.20	7048.40	0.00	0.00

8	Network	0.00	0.00	18,585.60	15,994.24	9926.40	8072.16	28,512.00	24,066.40	193,536.00	168,089.60
	Av. Per Node	0.00	0.00	232.32	199.93	124.08	100.90	356.40	300.83	2419.20	2101.12
	Worst Case (32)	0.00	0.00	1267.20	1350.72	624.00	423.44	1891.20	1774.16	12,902.40	13,807.36

14	Network	0.00	0.00	18,585.60	15,994.24	9705.60	7809.52	28,291.20	23,803.76	193,536.00	168,089.60
	Av. Per Node	0.00	0.00	232.32	199.93	121.32	97.62	353.64	297.55	2419.20	2101.12
	Worst Case (50)	0.00	0.00	1910.40	2068.96	336.00	230.48	2246.40	2299.44	19,353.60	21,011.20

20	Network	768.00	3044.48	18,585.60	15,994.24	10,771.20	8961.92	30,124.80	28,000.64	145,152.00	126,067.20
	Av. Per Node	9.60	38.06	232.32	199.93	134.64	112.02	376.56	350.01	1814.40	1575.84
	Worst Case (32)	9.60	42.88	1382.40	1479.36	710.40	493.12	2102.40	2015.36	10,483.20	11,256.00

**Table 7.** Bytes Transmitted and Received with PBC Authentication.

**Pass**		**Hello**	**Reports**	**SUB**	**Total OH**	**Normal**	**TOTAL**
2	Network	14,608.00	476,434.00	8030.00	499,072.00	0.00	499,072.00
	Av. Per Node	182.60	5955.43	100.38	6238.40	0.00	6238.40
	Worst Case (42)	220.00	6663.00	426.00	7309.00	0.00	7309.00

8	Network	0.00	19,656.00	8274.00	27,930.00	122,880.00	150,810.00
	Av. Per Node	0.00	245.70	103.43	349.13	1536.00	1885.13
	Worst Case (32)	0.00	1456.00	489.00	1945.00	9024.00	10,969.00

14	Network	0.00	19,656.00	7939.00	27,595.00	122,880.00	150,475.00
	Av. Per Node	0.00	245.70	99.24	344.94	1536.00	1880.94
	Worst Case (50)	0.00	2204.00	273.00	2477.00	13,632.00	16,109.00

20	Network	8008.00	19,656.00	9476.00	37,140.00	92,160.00	129,300.00
	Av. Per Node	100.10	245.70	118.45	464.25	1152.00	1616.25
	Worst Case (32)	110.00	1584.00	600.00	2294.00	7344.00	9638.00

**Table 8.** Transmission Energy in (μJ) with PBC Authentication.

**Pass**		**Hello**		**Reports**		**SUB**		**OH**		**Normal**	

		**tx**	**rx**	**tx**	**rx**	**tx**	**rx**	**tx**	**rx**	**tx**	**Rx**
2	Network	38,438.40	35,376.00	758,342.40	1,706,870.56	19,564.80	21,193.44	816,345.60	1,763,440	0.00	0.00
	Av. Per Node	480.48	442.20	9479.28	21,335.88	244.56	264.92	10,204.32	22,043.00	0.00	0.00
	Worst Case (42)	528.00	589.60	10,310.40	24,200.40	1132.80	1018.40	11,971.20	25,808.40	0.00	0.00

8	Network	0.00	0.00	53,145.60	46,010.24	20,102.40	21,900.96	73,248	67,911.20	331,776.	288,153.60
	Av. Per Node	0.00	0.00	664.32	575.13	251.28	273.76	915.60	848.89	4147.20	3601.92
	Worst Case (32)	0.00	0.00	3571.20	3816.32	1296.00	1173.84	4867.20	4990.16	22,118.40	23,669.76

14	Network	0.00	0.00	53,145.60	46,010.24	19,401.60	20,887.92	72,547.20	66,898.16	331,776	288,153.60
	Av. Per Node	0.00	0.00	664.32	575.13	242.52	261.10	906.84	836.23	4147.20	3601.92
	Worst Case (50)	0.00	0.00	5366.40	5820.96	720.00	659.28	6086.40	6480.24	33,177.60	36,019.20

20	Network	8448.00	33,489.28	53,145.60	46,010.24	22,771.20	25,363.52	84,364.80	104,863.04	248,832	216,115.20
	Av. Per Node	105.60	418.62	664.32	575.13	284.64	317.04	1054.56	1310.79	3110.40	2701.44
	Worst Case (32)	105.60	471.68	3878.40	4159.36	1574.40	1457.92	5558.40	6088.96	17,971.20	19,296.00

**Table 9.** Total Number of Authenticated Bytes.

**Pass**		**SIGN**	**VERIFY**	**ENC**	**DEC**	**TOTAL**
2	Network	1340.00	132,280.00	0.00	2040.00	135,660.00
	Av. Per Node	16.75	1653.50	0.00	25.50	1695.75
	Worst Case (42)	2029.00	2029.00	7309.00	7309.00	18,676.00

8	Network	9848.00	37,956.00	22,400.00	18,440.00	88,644.00
	Av. Per Node	123.10	474.45	280.00	230.50	1108.05
	Worst Case (32)	5989.00	725.00	10,969.00	1945.00	19,628.00

14	Network	9848.00	37,760.00	22,400.00	18,340.00	88,348.00
	Av. Per Node	123.10	472.00	280.00	229.25	1104.35
	Worst Case (50)	8849.00	897.00	16,109.00	2477.00	28,332.00

20	Network	7768.00	31,356.00	16,800.00	14,740.00	70,664.00
	Av. Per Node	97.10	391.95	210.00	184.25	883.30
	Worst Case (32)	5098.00	814.00	9638.00	2294.00	17,844.00

**Table 10.** Cumulative Number of Bytes Up to each Pass.

**Cumulative Number of Bytes Up to each Pass**						

**Pass**			**2**	**8**	**14**	**20**
Broadcasting Rate without authentication	10	Network	134,630.00	367,907.00	601,184.00	834,461.00
		Av. Per Node	1682.88	4598.84	7514.80	10,430.76
		Worst Case	1973.00	12,719.00	23,465.00	34,211.00

	50	Network	136,404.00	465,790.00	795,176.00	1,124,562.00
		Av. Per Node	1705.05	5822.38	9939.70	14,057.03
		Worst Case	2075.00	21,463.00	40,851.00	60,239.00

	100	Network	135,368.00	569,250.00	1,003,132.00	1,437,014.00
		Av. Per Node	1692.10	55,927.35	110,162.60	164,397.85
		Worst Case	1997.00	34,356.00	66,715.00	99,074.00

Normal Case w/auth		Network	499,072.00	735,793.00	886,268.00	1,015,568.00
		Av. Per Node	6238.40	9197.41	11,078.35	12,694.60
		Worst Case	7309.00	25,543.00	41,652.00	51,290.00

**Table 11.** Network Life-time Calculations.

**X**	**10%**		**50%**		**100%**	

	**First Pass**	**Av**	**First Pass**	**Av**	**First Pass**	**Av**
Energy Node Av. (mJ)	33.16	9.61	36.79	11.83	41.34	14.59
Energy Worst Case (mJ)	47.79	73.86	87.83	120.89	137.88	179.68

Av. Node Dead (Passes)	1,560,206.07		1,268,336.34		1,027,959.17	
FND (Passes)	203,073.02		124,076.20		83,482.07	

## References

[1] Akyildiz I.F., Su W., Sankarasubramaniam Y., Cayirci E. (2002). A Survey on Sensor Networks. IEEE Commun. Mag..

[2] Karlof C., Wagner D. (2003). Secure routing in wireless sensor networks: Attacks and countermeasures. Ad Hoc Netw..

[3] Srinivasan A., Teitelbaum J., Wu J., Cardei M., Liang H., Boukerche A. (2009). Reputation and Trust-based Systems for Ad Hoc and Sensor Networks. Algorithms and Protocols for Wireless, Mobile Ad Hoc Networks.

[4] Tanachaiwiwat S., Dave P., Bhindwale R., Helmy A. Location-centric Isolation of Misbehavior and Trust Routing in Energy-constrained Sensor Networks.

[5] Huang L., Li L., Tan Q. (2006). Behavior-Based Trust in Wireless Sensor Network. Adv. Web Netw. Technol. Appl. LNCS.

[6] Tajeddine A., Chehab A., Kayssi A. CENTER: A Centralized Trust-Based Efficient Routing Protocol for Wireless Sensor Networks.

[7] Al-Karaki J., Kamal A. (2004). Routing Techniques in Wireless Sensor Networks: A Survey. IEEE Wirel. Commun..

[8] Schurgers C., Srivastava M. Energy Efficient Routing in Wireless Sensor Network.

[9] Momani M., Challa S., Aboura K. (2007). Modelling Trust in Wireless Sensor Networks from the Sensor Reliability Prospective. Innov. Algorithms Tech. Ind. Electron. Telecommun..

[10] Yadav K., Srinivasan A. iTrust: An Integrated Trust Framework for Wireless Sensor Networks.

[11] Momani M. (2010). Trust Models in Wireless Sensor Networks: A Survey. Recent Trends Netw. Secur. Appl..

[12] Chen X., Makki K., Yen K., Pissinou N. (2009). Sensor Network Security: A Survey. IEEE Commun. Surveys Tutor..

[13] Yu Y., Li K., Zhou W., Li P. (2012). Trust mechanisms in wireless sensor networks: Attack analysis and countermeasures. J. Netw. Comput. Appl..

[14] Muruganathan S., Ma D., Bhasin R., Fapojuwo A. (2005). A Centralized Energy-Efficient Routing Protocol for Wireless Sensor Networks. IEEE Radio Commun..

[15] Akkaya K., Younis M. (2005). A survey on routing protocols for wireless sensor networks. Ad Hoc Netw..

[16] Manjeshwar A., Agrawal D.P. TEEN: A routing protocol for enhanced efficiency in wireless sensor networks.

[17] Manjeshwar A., Agrawal D.P. APTEEN: A hybrid protocol for efficient routing and comprehensive information retrieval in wireless sensor networks.

[18] Chang J.H., Tassiulas L. (2004). Maximum lifetime routing in wireless sensor networks. IEEE/ACM Trans. Netw. (TON).

[19] Yu Y., Govindan R., Estrin D. (2001). Geographical and Energy Aware Routing: A Recursive Data Dissemination Protocol for Wireless Sensor Networks.

[20] Akyildiz I.F., Su W., Sankarasubramaniam Y., Cayirci E. (2002). Wireless sensor networks: A survey. Comput. Netw..

[21] Ganesan D., Govindan R., Shenker S., Estrin D. (2001). Highly-resilient, energy-efficient multipath routing in wireless sensor networks. ACM SIGMOBILE Mob. Comput. Commun. Rev..

[22] ONF Market Education Committee Software-defined networking: The new norm for networks. ONF White Paper (2012). https://www.opennetworking.org/images/stories/downloads/sdn-resources/white-papers/wp-sdn-newnorm.pdf.

[23] Tajeddine A., Kayssi A., Chehab A., Elhajj I. Authentication Schemes for Wireless Sensor Networks.

[24] Mani D., Nishamol P. (2013). A Comparison between RSA and ECC in Wireless Sensor Networks. Int. J. Eng. Res. Technol..

[25] TinyOS http://www.tinyos.net/.

[26] TinyPairing: A Pairing-Based Cryptographic Library for Wireless Sensor Networks. http://www.cs.cityu.edu.hk/~ecc/TinyPairing/.

[27] Xiong X., Wong D.S., Deng X. TinyPairing: A Fast and Lightweight Pairing-based Cryptographic Library for Wireless Sensor Networks.

[28] Meulenaer G.D., Gosset F., Standaert F.X., Pereira O. On the energy cost of communication and cryptography in wireless sensor networks.

